# Gold(I)-Catalyzed Intermolecular Reactions of Alkynes with Carbon Nucleophiles: What We Know—and What We Don't

**DOI:** 10.1021/acscatal.6c00792

**Published:** 2026-03-30

**Authors:** Jesús Rodrigalvarez, Anna Arnanz, Gala Ogalla, Antonio M. Echavarren

**Affiliations:** † Institute of Chemical Research of Catalonia (ICIQ-CERCA), Barcelona Institute of Science and Technology, Av. Països Catalans 16, Tarragona 43007, Spain; ‡ Departament de Química Orgànica i Analítica, Universitat Rovira i Virgili, C/Marcel·li Domingo s/n, Tarragona 43007, Spain

**Keywords:** gold catalysis, alkynes, alkenes, gold carbenes, cycloadditions, rearrangements

## Abstract

We present an overview of the scope, limitations, and challenges associated with gold­(I)-catalyzed intermolecular reactions involving alkynes and alkenes, or alkynes with other alkynes, as well as mechanistically related transformations with a broader range of carbon-based nucleophiles. Emphasis is placed on reactivity patterns, selectivity issues, and the factors that currently limit the generality and synthetic applicability of these processes.

## Graphical abstract

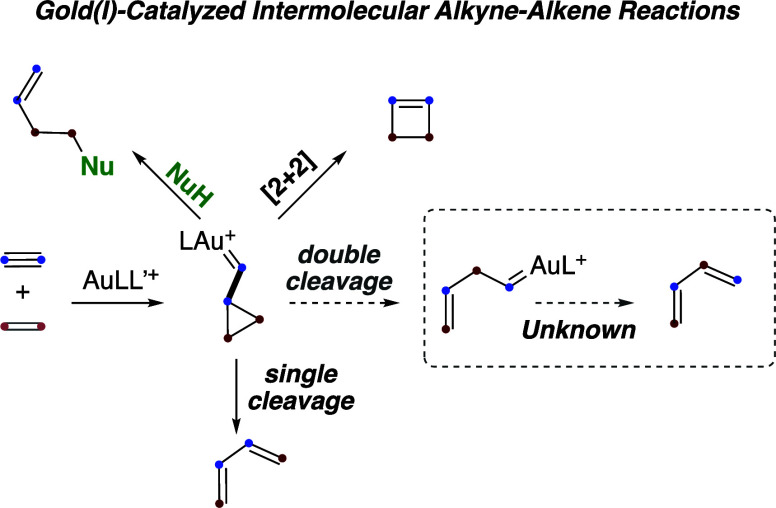

## Introduction

The field of homogeneous gold catalysis emerged with the discovery that cationic phosphine-gold­(I) complexes are highly active catalysts for the addition of alcohols to alkynes, leading to the formation of acetals or enol ethers.^1^ Shortly thereafter, the gold­(I)-catalyzed addition of water to alkynes was reported.^2^ These seminal discoveries triggered pioneering studies on the cyclization of 1,5- ^3,4^ and 1,6-enynes,^5,6^ as well as closely related substrates.^7^ The intramolecular reaction of furans with alkynes,^8,9^ which was originally reported as a gold­(III)-catalyzed process, was later shown to be mechanistically related to gold­(I)-catalyzed enyne cyclizations.^10,11^ These early findings laid the foundation for the development of a range of cyclization reactions involving alkyne-containing substrates.^12–19^

However, the development of broadly applicable intermolecular gold­(I)-catalyzed reactions between alkynes and alkenes has progressed much more slowly. In addition to the inherent entropic disadvantages compared to intramolecular processes, a major challenge arises from the fact that the products of these intermolecular reactions are themselves alkenes, which can subsequently react with alkynes, leading to oligomer formation.

Herein, we discuss the principal gold­(I)-catalyzed intermolecular reactions between alkynes and alkenes as well as related transformations involving other carbon-based nucleophiles, with particular emphasis on their mechanistic features. We also briefly address the current limitations and remaining gaps in synthetic methodology within this area of gold­(I) catalysis.

## Reaction of Alkynes with Alkenes via Cyclopropyl Gold(I) Carbenes

The gold­(I)-catalyzed cyclization of 1,*n*-enynes proceeds via η^2^-alkyne gold­(I) complexes of type **1**, which form cyclopropyl gold­(I) carbenes **2a** (Scheme 1).^5,6,17^ These intermediates can subsequently be trapped by alcohols, water, or other nucleophiles, ultimately affording adducts of type **3**. In a few cases, cyclobutenes **4** have been obtained in the gold­(I)-catalyzed cycloisomerizations.^20–22^ Intermediates **2a** can also form *exo*-type 1,3-dienes **5a** and/or *endo*-type 1,3-dienes **5b** by single-cleavage rearrangement.^23–28^ As an alternative, double-cleavage rearrangement of **2a** can compete to form gold­(I) carbene intermediates **6**, which undergo α-proton elimination to afford 1,3-dienes **7**.

**Scheme 1:**
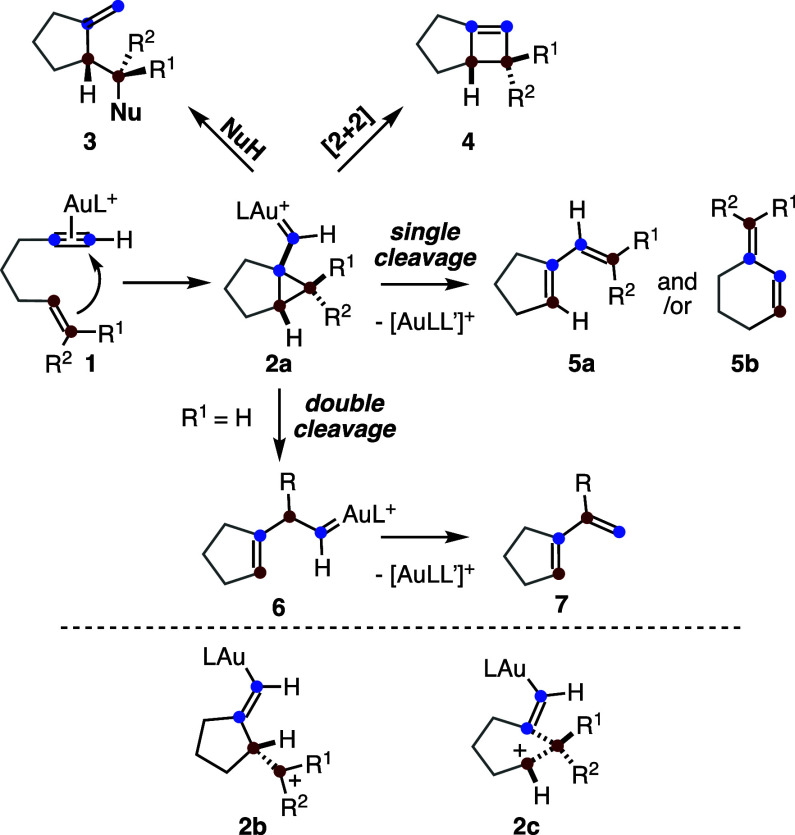
Gold­(I)-Catalyzed Addition of Nucleophiles to 1,*n*-Enynes vs Cycloaddition or Skeletal Rearrangements

Although the key intermediates in this type of cyclization are often depicted as cyclopropyl gold­(I) carbenes **2a**, computational studies have revealed a more complex scenario in which, in addition to **2a**, cationic species **2b** and **2c** may also be in equilibrium with **1a**.^26,27^

The intermolecular reaction of terminal alkynes **8** with alkenes leads to the selective formation of cyclobutenes **9** by a formal [2 + 2] cycloaddition (Scheme 2).^29–31^ The observed regioselectivity can be explained by the Markovnikov addition of the cationic alkyne-gold­(I) complex to the alkene, leading to distorted cyclopropyl gold­(I) carbenes of type **10**.^26–28^ Electron-rich alkynes, primarily aryl alkynes as well as heteroaryl, alkenyl, and 1,3-enynes, serve as suitable partners in these cycloaddition reactions. Except for cyclopropylacetylene, aliphatic alkynes generally fail to deliver the desired cyclobutenes in good yields. On the other hand, while monosubstituted alkenes are unreactive, di- to tetrasubstituted alkenes, including 1,3-enynes and 1,3-dienes,^31^ participate efficiently in the transformation.

**Scheme 2:**
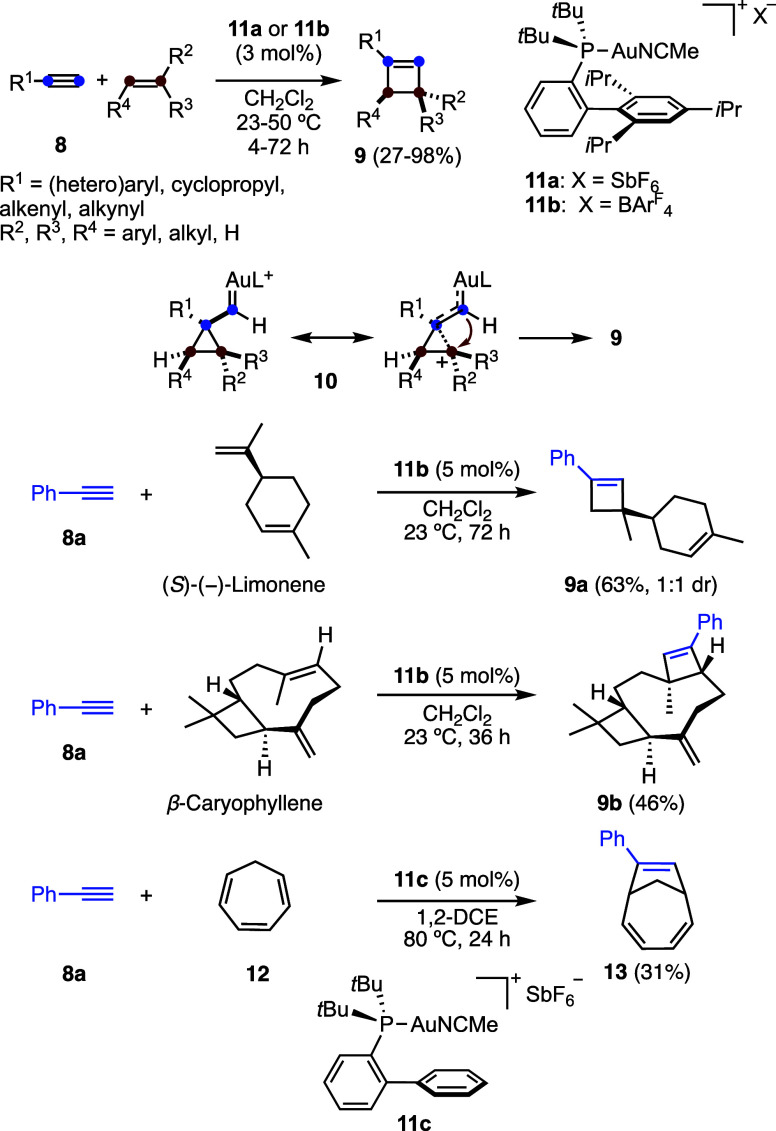
Gold­(I)-Catalyzed [2 + 2] Cycloaddition of Terminal Alkynes with Alkenes

As examples of site-selectivity in the reaction with natural products containing 1,5-dienes,^31^ phenylacetylene (**8a**) undergoes cycloaddition at the most accessible disubstituted terminal alkene with limonene to afford adduct **9a**, whereas **8a** reacts with β-caryophyllene at the more electron-rich trisubstituted alkene to give **9b**. As an interesting exception, the gold­(I)-catalyzed reaction of **8a** with cycloheptatriene (**12**) led instead to **13**, a product of a formal [6 + 2] cycloaddition.^31^

The use of gold­(I) complexes such as **11a**–**b** with bulky biphenylphosphine ligands was key to the success of the [2 + 2] cycloaddition.^29–31^ In another study, s,p-alkynyl digold­(I)-complexes were detected in the reaction, which were catalytically active.^32^ This type of dinuclear complex was also detected and characterized in other gold­(I)-catalyzed reactions.^33^

As mentioned before, cyclobutenes have been obtained as the major products in a few gold­(I)-catalyzed cycloisomerizations. For example, reactions of 1,7-enynes such as **14a**–**b** give rise to products **15a**–**b** (Scheme 3).^20^ Notably, even certain 1,6-enynes have been shown to undergo cycloisomerization to afford highly strained bicyclo[3.2.0]­hept-5-enes, featuring a bicyclic skeleton embedded within a *trans*-cycloheptene.^20^

**Scheme 3:**
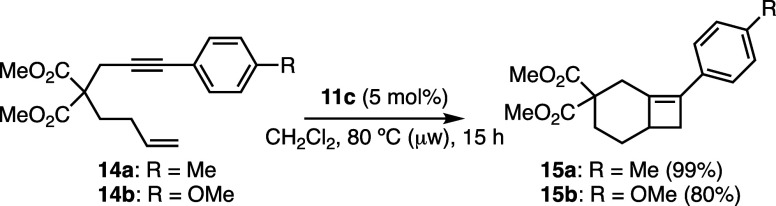
Intramolecular Gold­(I)-Catalyzed [2 + 2] Cycloaddition

The enantioselective [2 + 2] cycloaddition was developed using monocationic Josiphos digold­(I) catalysts derived from complexes such as **16a**–**b** (Scheme 4).^34^ Up to 94:6 *er* were achieved, although in most cases, the enantiomeric ratios were around 85:15. Importantly, only the gold­(I) center with the trialkylphosphine ligand is directly involved in alkyne activation, whereas the second metal center is found to be essential for inducing enantioselectivity. This enantioselective cycloaddition was applied to a relatively concise enantioselective synthesis of the cyclobutene natural product rumphellaone A (**19**) via reaction of **8a** with alkene **17** to form cyclobutene **18** using Josiphos digold­(I) catalyst **16a** (Scheme 5).^34^ An alternative total synthesis of rumphellaone A (**19**) was also developed using an intramolecular [2 + 2] cycloaddition of a 1,10-enyne.^22^

**Scheme 4:**
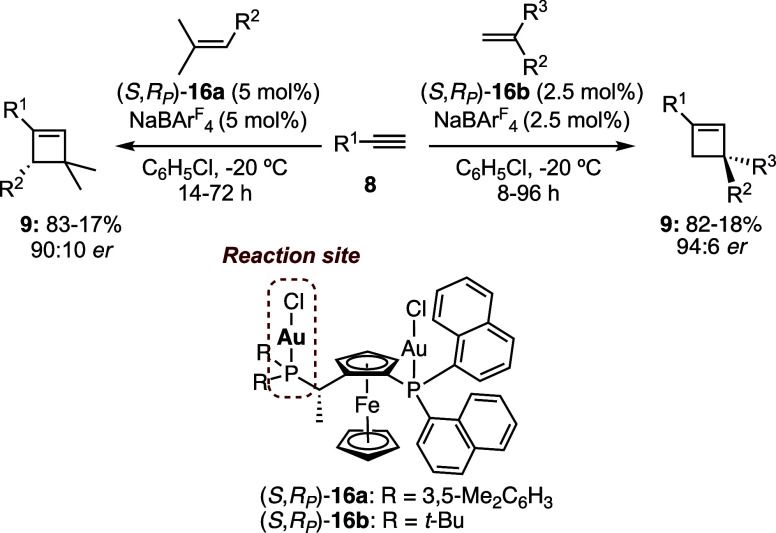
Enantioselective Gold­(I)-Catalyzed [2 + 2] Cycloaddition to Form Cyclobutenes

**Scheme 5:**
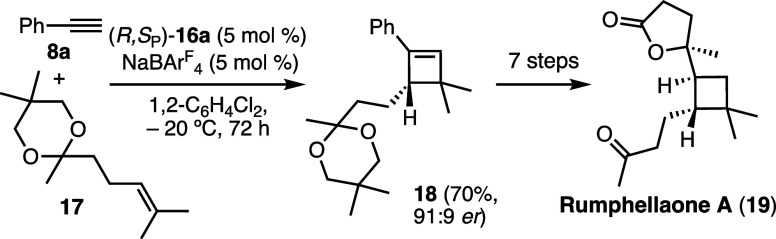
Enantioselective Gold­(I)-Catalyzed Total Synthesis of Rumphellaone A

1,3-Butadienes were observed as secondary products in the gold­(I)-catalyzed [2 + 2] cycloaddition between alkynes and alkenes.^30^ For example, *o*-fluorophenylacetylene (**8b**) reacts with α-methylstyrene to give the expected cyclobutene **9c** in 64% yield, together with trace amounts of 1,3-diene **20a** (3%) (Scheme 6a). In contrast, *o*-chloro (**8c**) and *o*-bromophenylacetylene (**8d**) predominantly yield the corresponding 1,3-dienes **20b** and **20c**, respectively. On the other hand, a separate study showed that the reaction of **8d** and *o*-iodophenylacetylene with 2,3-dimethyl-2-butene affords only the corresponding cyclobutene.^35^

**Scheme 6:**
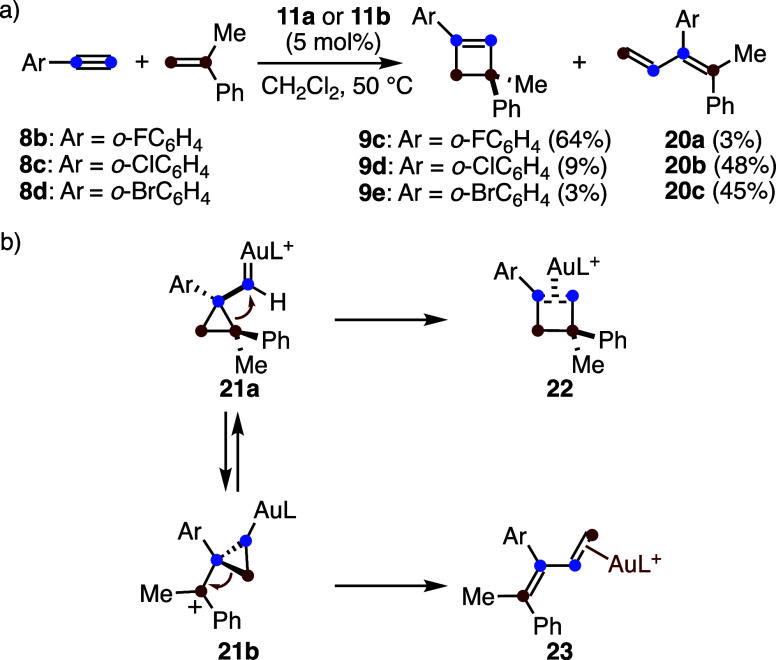
(a) Formation of 1,3-Dienes in the Intermolecular Reaction of Alkynes with Alkenes. (b) Mechanism for the Formation of Cyclobutenes or 1,3-Dienes

The formation of both cyclobutenes and 1,3-dienes in the gold­(I)-catalyzed intermolecular reaction of alkynes with alkenes can be mechanistically rationalized by the initial formation of cyclopropyl gold­(I) carbene **21a** (Scheme 6b).^30^ This species may either undergo ring expansion to give the cyclobutene-gold­(I) complex **22** or rearrange to form intermediate **21b**, which subsequently undergoes opening to afford 1,3-butadiene-gold­(I) complex **23**. According to DFT calculations, differences in the activation energies for the expansion or rearrangement of cyclopropyl gold­(I) carbenes **21** are small, so subtle changes in the substitution pattern of the substrates modify the steric interactions and, consequently, the reaction outcome.

It is important to note that, although 1,3-dienes **20** may be regarded as formal products of alkyne/alkene metathesis, the gold­(I)-catalyzed reaction is fundamentally different from alkyne/alkene metathesis initiated by [2 + 2] cycloaddition of metal carbenes, such as Grubbs-type carbenes. In the latter processes, the initial metal carbene reacts with an alkene to generate a new carbene, which subsequently undergoes an intramolecular reaction with the alkyne to form the final 1,3-diene.^36^ In that case, each catalytic cycle incorporates a carbon atom from a different molecule, whereas the gold­(I)-catalyzed metathesis-type reaction shown in Scheme 6 is an entirely intramolecular process.

Intramolecular trapping of cyclopropyl gold­(I) carbene intermediates takes place in the formal [2 + 2 + 2] cycloaddition of terminal alkynes **8** with oxoalkenes **24**, leading to the formation of oxabicyclo[3.2.1]­oct-3-enes **25** through the construction of two C–C bonds and one C–O bond (Scheme 7a).^33^ This transformation proceeds by intramolecular reaction of cyclopropyl gold­(I) carbene intermediate **26** with the carbonyl group to form oxonium species **27**, which subsequently undergoes an intramolecular Prins-type reaction to form the final C–C bond, affording product **28**. Cyclobutenes became the major products with substrates bearing more electron-rich alkenes. For example, the reaction of alkyne **8a** with oxoalkene **24b** affords a 4:1 mixture of cyclobutene **9f** and a seven-membered ring product **25b**.

**Scheme 7:**
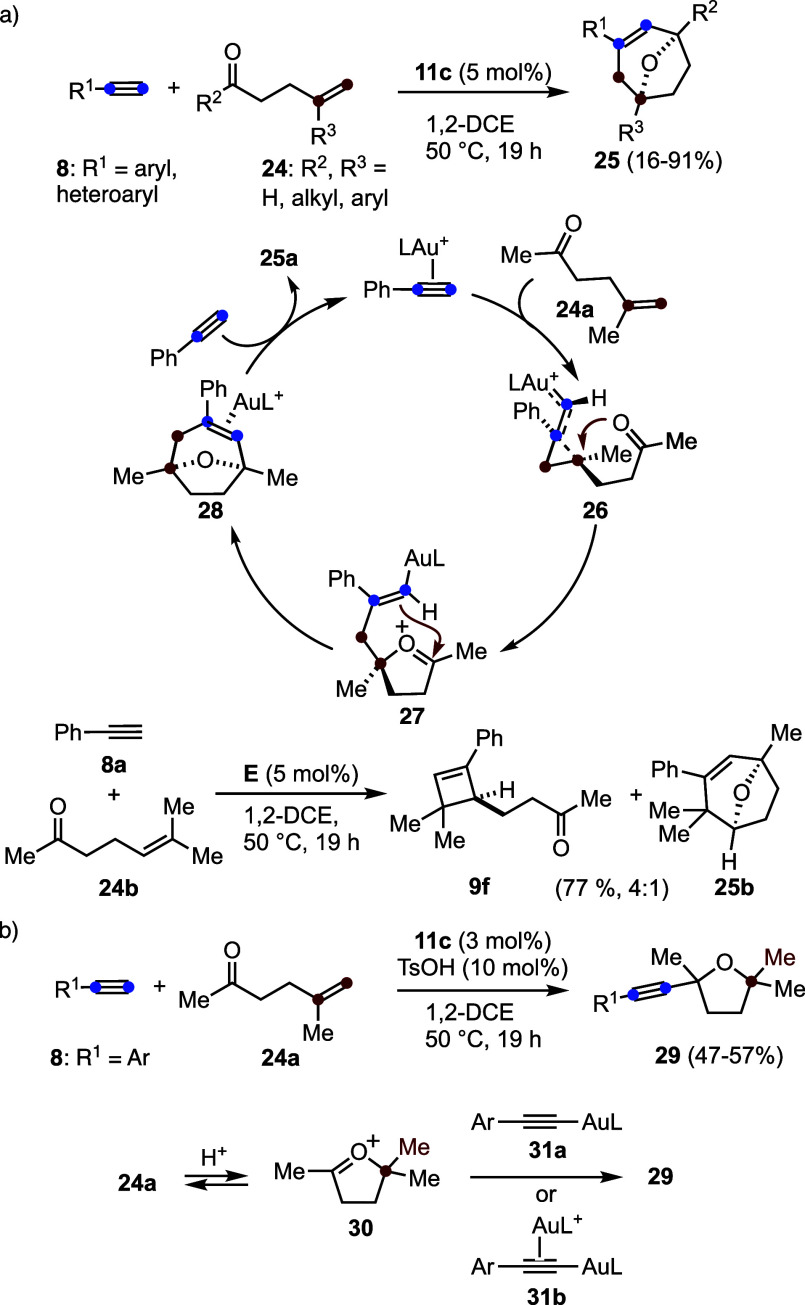
(a) Formal Gold­(I)-Catalyzed [2 + 2 + 2] Cycloaddition of Alkynes with Oxoalkenes. (b) Gold­(I)- and Bro̷nsted Acid-Catalyzed Formation of Tetrahydrofurans

Performing the gold­(I)-catalyzed reaction in the presence of TsOH as a Bro̷nsted acid and cocatalyst leads to the formation of tetrahydrofurans **29** (Scheme 7b).^33^ This transformation is proposed to proceed via oxonium cations **30**, which are intercepted by σ-alkynyl-gold­(I) complexes **31a** or σ,π-alknyl digold­(I)-complexes **31b** acting as nucleophiles. Notably, digold­(I) complexes **31b** are, on their own, catalytically inactive, as no reaction was observed under stoichiometric conditions. Consistent with this observation, under catalytic conditions employing 2.5 mol % complex **31b** (Ar = Ph, L = JohnPhos), only 9% of **25a** was obtained after 19 h at 50 °C.

Chloroalkynes also undergo [2 + 2] cycloaddition reaction with alkenes (Scheme 8).^37^ (Chloroethynyl)­arenes **32a** and phenyl chloroethynyl sulfide **32b** react with electronically nonactivated alkenes, ranging from monosubstituted to symmetric disubstituted alkenes to afford cyclobutenes **33**. In the case of symmetrically disubstituted alkenes, the reaction was shown to be largely stereospecific.

**Scheme 8:**
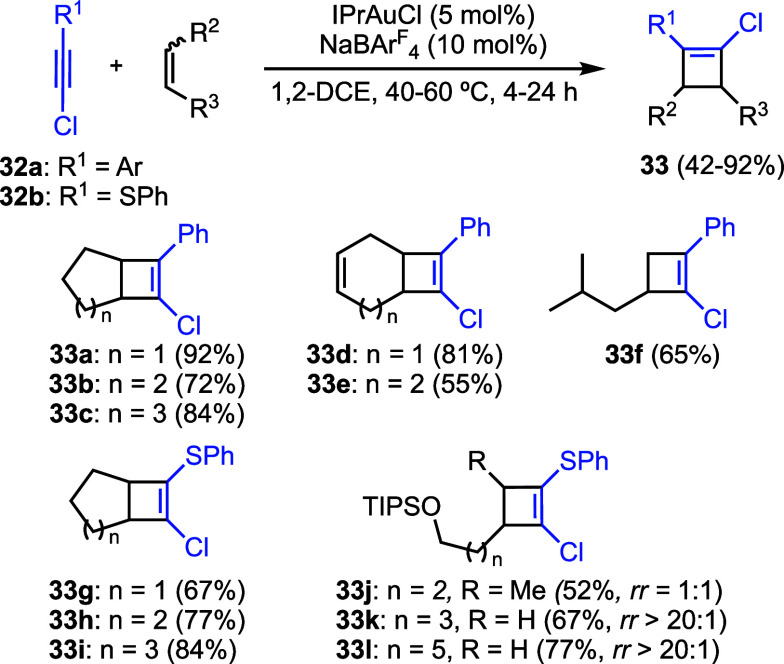
Gold­(I)-Catalyzed [2 + 2] Cycloaddition of Chloroalkynes with Alkenes[Fn sch8-fn1]

a
*rr* = regioisomeric ratio.

In a similar vein, the gold­(I)-catalyzed [2 + 2] cycloaddition of arylynol ethers **34** with alkenes proceeds regioselectively to form cyclobutenes **35**, which can easily be converted into cyclobutanones **36** (Scheme 9).^38^ This is, therefore, complementary to the well established synthesis of cyclobutanones by [2 + 2] cycloaddition of ketenes with alkenes.^39,40^

**Scheme 9:**
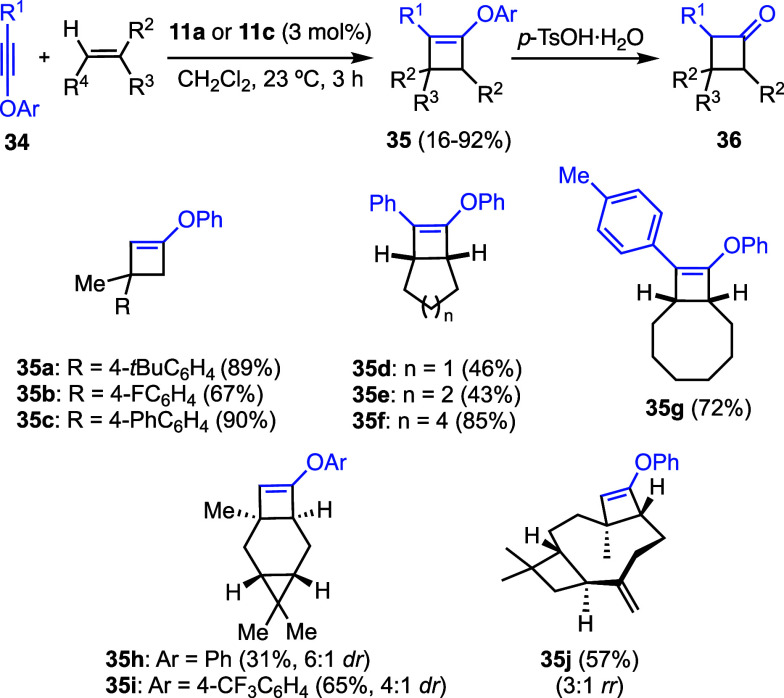
Gold­(I)-Catalyzed [2 + 2] Cycloaddition of Ynol Ethers with Alkenes

Interestingly, internal arylynol ethers **34a** react with styrenes via formal (4 + 2) cycloaddition to form **37a** proceeding via cyclopropyl gold­(I) carbene intermediates **38** (Scheme 10a).^38^ A related formal (4 + 2) cycloaddition was found in the gold­(I)-catalyzed reaction of ynol ethers **34b** with enol ethers leading to products **37b** (Scheme 10b).^41^

**Scheme 10:**
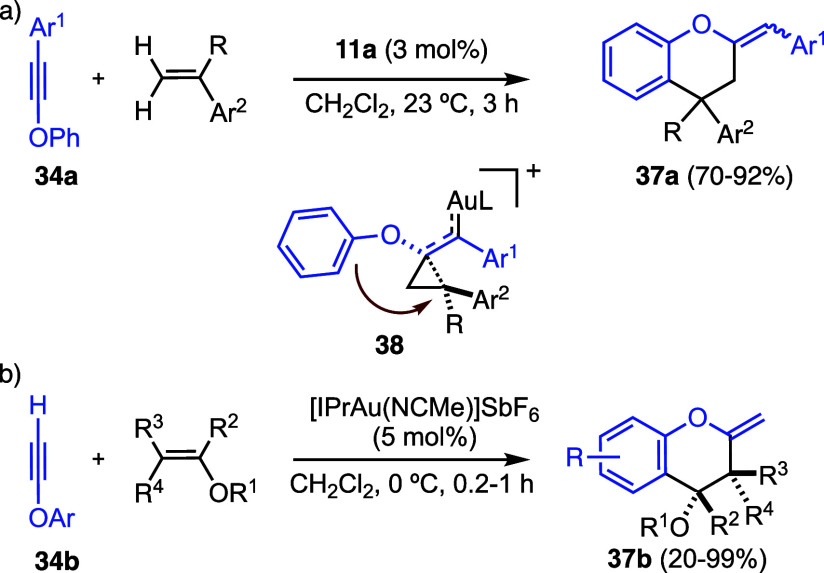
(a) Formal Gold­(I)-Catalyzed (4 + 2) Cycloaddition of Ynol Ethers with Alkenes. (b) Formal Gold­(I)-Catalyzed (4 + 2) Cycloaddition of Ynol Ethers with Enol Ethers

The reaction of propylic acid (**39a**) and *t*-butyl propiolate (**39b**) leads to the formation of lactones **40** (Scheme 11).^42,43^ Notably, *t*-butyl propiolate (**39b**) is effectively equivalent to propylic acid (**39a**), as under the action of Lewis-acidic gold­(I) catalysts, **39b** is converted into **39a** with the release of isobutylene.^42^ As an example, the reaction of **39b** with *E*-1-phenyl-1,3-butadiene afforded the natural product goniothalamin (**40c**) in racemic form by regioselective addition to the less substituted double bond of the 1,3-diene. Remarkably, a 1,3-disubstituted allene could also be used as a cycloaddition partner to furnish lactone **40e**, in a rare example of an intermolecular alkyne-allene gold­(I)-catalyzed reaction. The enantioselective (4 + 2) annulation of propiolates and alkenes to afford α,β-unsaturated lactones **40** was achieved using a chiral digold­(I) catalyst in 1,1,2,2-tetrachloroethane (TCE) as solvent and sodium dodecyl sulfate (SDS) as an anionic surfactant, which was shown to improve the selectivity.^44^

**Scheme 11:**
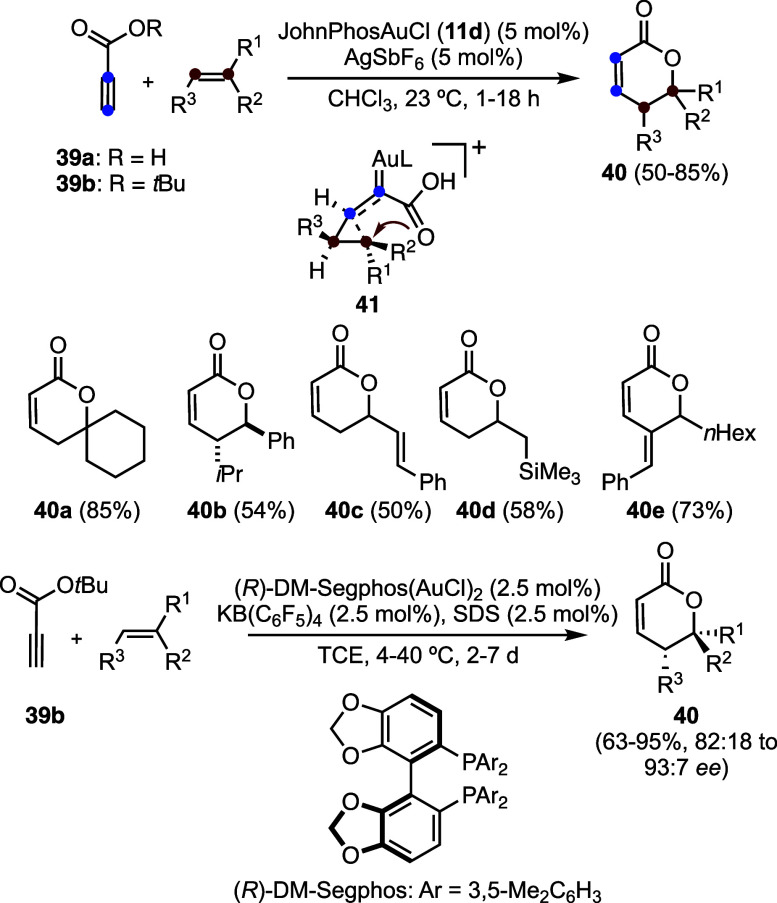
Gold­(I)-Catalyzed Reaction of Propiolic Acid and Related Alkynes with Alkenes to Form α,β-Unsaturated Lactones

The reaction of propiolic acid (**39a**) and related alkynes **39b**–**e** with 1,2-disubstituted alkenes results in the formation of (*E*,*Z*)-1,3-butadienes **42** by a rearrangement process (Scheme 12).^42,43^ From a synthetic standpoint, this transformation is particularly attractive as it enables the expansion of cyclic alkenes by two carbon units. For instance, cycloheptene is converted into nine-membered ring 1,3-diene **42c**, while cyclooctene affords ten-membered ring **42d**. Mechanistically, this transformation closely parallels the formation of 1,3-butadienes **20** observed in the reaction of arylalkynes with electron-rich alkenes (see Scheme 6a).^30^ Computational studies support a similar pathway in which the initially generated cyclopropyl gold­(I) carbene **43** undergoes isomerization to form cyclopropylmethyl cation **44**, which subsequently undergoes stereoselective ring opening to give 1,3-diene-gold­(I) complexes **45**.

**Scheme 12:**
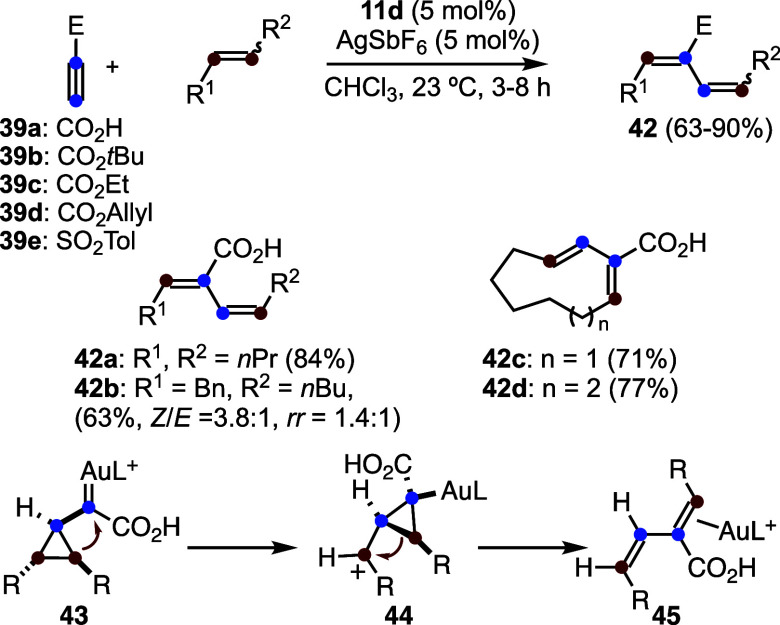
Gold­(I)-Catalyzed Reaction of Propiolic Acid and Related Alkynes with Alkenes to Afford 1,3-Butadienes by Rearrangement

The gold­(I)-catalyzed intermolecular reaction of alkynoates such as **39f** with allylic ethers **46** affords *b*-alkoxyacrylates **47** (Scheme 13a).^43,45^ This transformation represents an exception in the general reactivity pattern of alkynes with alkenes, which typically proceeds through the formation of cyclopropyl gold­(I) carbenes as intermediates. In this case, gold­(I) instead promotes the nucleophilic addition of the ether to the activated alkyne, generating intermediate **48**, which subsequently undergoes a sigmatropic Claisen rearrangement to form **49**. Related reactivity has been observed with sulfonyl alkynes, although in those systems, a [1,3]-sigmatropic rearrangement was found to occur alongside with the [3,3]-pathway.^46^ Building on these findings, an enantioselective gold­(I)-catalyzed thioallylation of propiolates with allyl sulfides was developed, proceeding via a sulfonium Claisen rearrangement (Scheme 13b).^47,48^ The best enantioselectivities for the formation of products of type **47b** were achieved using the (*R*,*S*_*P*_)-enantiomer of Josiphos digold­(I) catalyst **16a** (see Scheme 4).

**Scheme 13:**
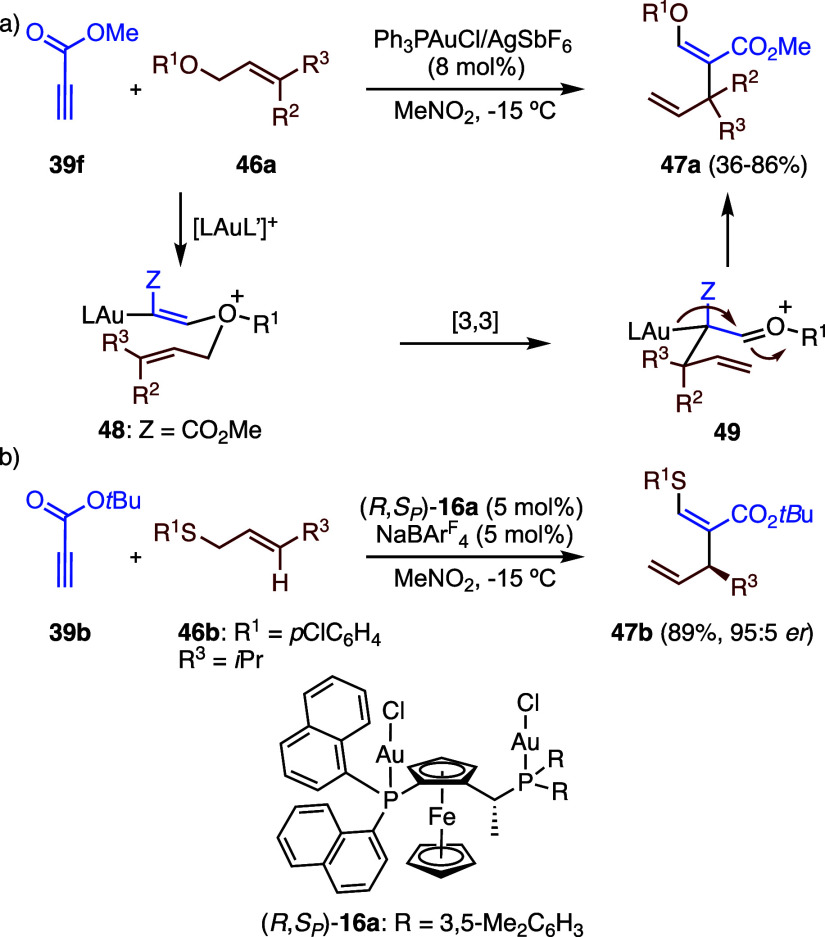
(a) Synthesis of β-Alkoxyacrylates by Gold­(I)-Catalyzed Reaction of Propiolic Esters with Allyl Ethers. (b) Enantioselective Gold­(I)-Catalyzed Thioallylation

The gold­(I)-catalyzed intermolecular reaction between acetylene gas and *trans*-stilbenes gives rise to (*Z*,*Z*)-configured 1,3-butadienes **50** by a metathesis-type process or biscyclopropanes **51** with a *meso*-configuration (Scheme 14).^49^ 1,3-Butadienes **50** arise by rearrangement of cyclopropyl gold­(I) carbenes **53**, as shown before in the formation of dienes **20** (Scheme 6)^30,31^ and **42** (Scheme 12).^42,43^ On the other hand, using catalysts such as **52**, with a more electron-donating NHC ligand, gives rise to a longer-lived intermediates **53**, which react intermolecularly with a second molecule of alkene to form biscyclopropanes **51**, instead of undergoing rearrangement. This reaction pathway and the *meso*-configuration, which was shown by X-ray diffraction, was supported by DFT calculations.^49^ All of these reactions are simply carried out under 1 atm of acetylene gas, which can be easily generated at room temperature by reaction of CaC_2_ with water for small scale operations.

**Scheme 14:**
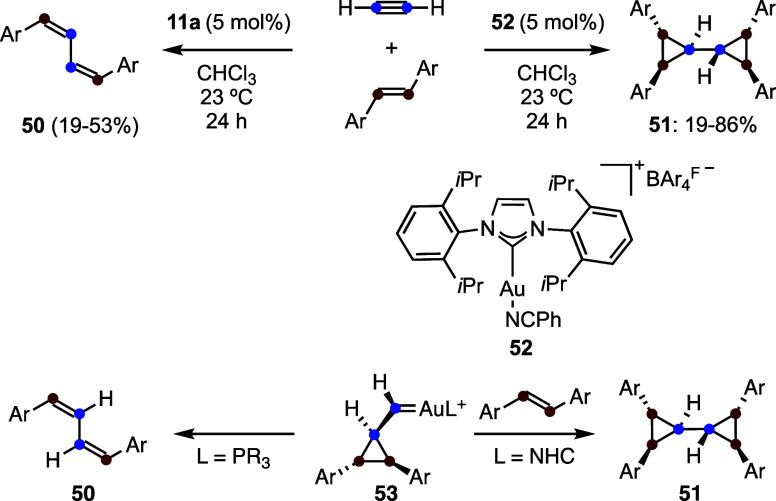
Rearrangement or Biscyclopropanation in the Gold­(I)-Catalyzed Reaction of Acetylene Gas

Experimental support for the rearrangement pathway leading to 1,3-butadienes was obtained by heating 7-cyclopropylcycloheptatriene **54** in the presence of gold­(I) catalyst **11a**, which resulted in the formation of *Z*,*Z*-1,4-diphenyl-1,3-butadiene (*Z*,*Z*-**50**) (Scheme 15).^30^ Decarbenation of **54** ^50–52^ generates the cyclopropyl gold­(I) carbene **53a**, which undergoes rearrangement to afford (*Z*,*Z*)-**50**. Notably, intermediate **53a** is identical to that generated in the gold­(I)-catalyzed reaction between acetylene and *trans*-stilbene. The alternative ring expansion of **53a** would have afforded cyclobutene **55**, whose conrotatory opening would have given diene *E*,*E*-1,4-diphenyl-1,3-butadiene (*E*,*E*-**50**),^53^ which was not experimentally observed.

**Scheme 15:**
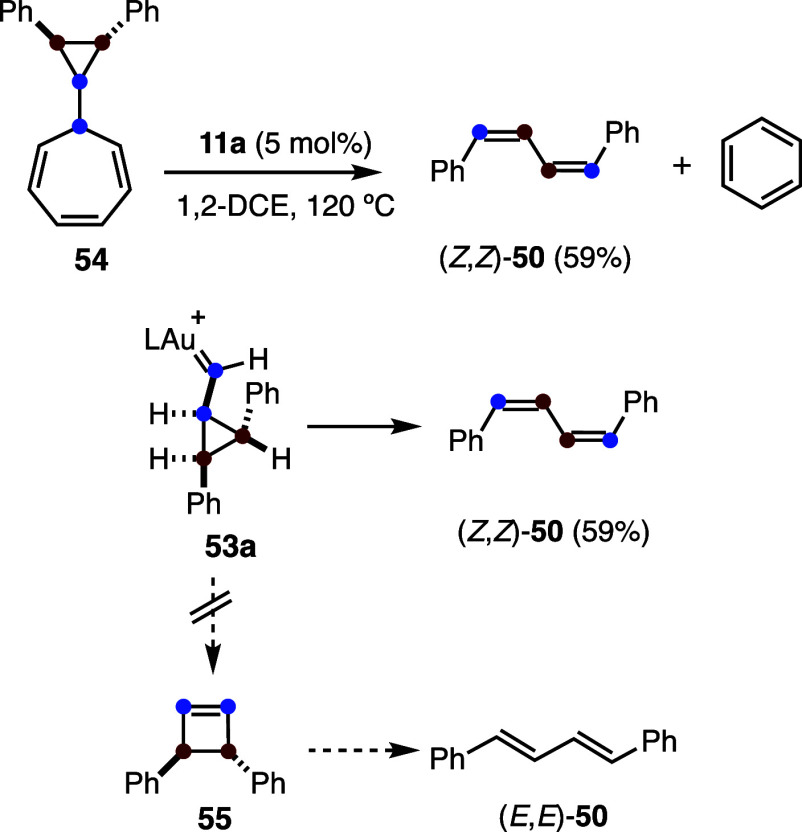
Decarbenation for the Generation of Cyclopropyl Gold­(I) Carbenes

Reaction of acetylene gas with 1,5-dienes **56** affords tricyclo­[5.1.0.0^2,4^]­octanes **57** as single diastereomers (Scheme 16).^49^ This biscyclopropanation strategy was applied in the first total synthesis of waitziacuminone (**59**), a sesquiterpene isolated from the Australian annual herb *Waitzia acuminata*, known as the orange immortelle. Specifically, the treatment of geranyl acetone (**58**) with acetylene gas in the presence of NHC-gold­(I) catalyst **52** delivered the natural product in a single step as a single diastereomer.

**Scheme 16:**
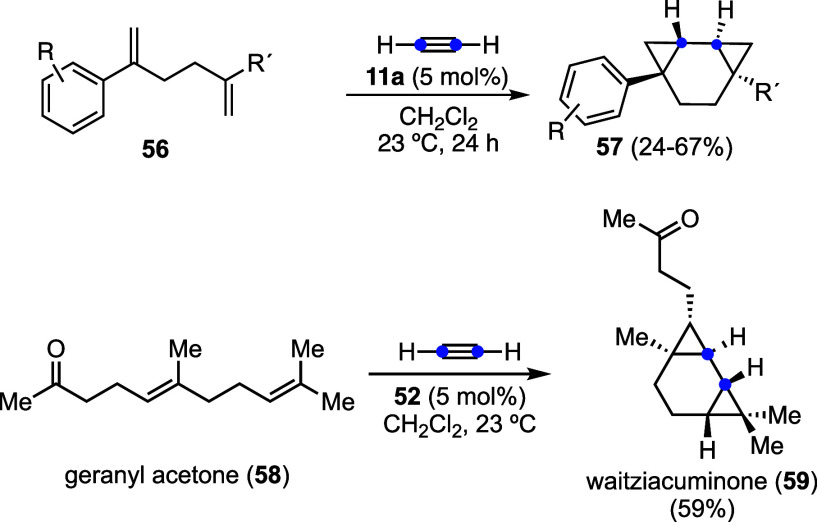
Synthesis of Tricyclo­[5.1.0.0^2,4^]­octanes and Total Synthesis of (±)-Waitziacuminone

The intermolecular gold­(I)-catalyzed reaction of *o*-allylphenols **60** with acetylene gas affords chromanes **61** via stereospecific aryloxycyclization, proceeding through nucleophilic regioselective ring opening of cyclopropyl gold­(I)-carbene intermediates **62** (Scheme 17).^54^ The synthetic utility of this methodology was demonstrated through the late-stage functionalization of natural product lapachol **63**, affording derivative **64**. Chromanes were obtained with moderate enantioselectivities (up to 83:17 *er*) using a JosiPhos-type digold­(I) complex as the catalyst.

**Scheme 17:**
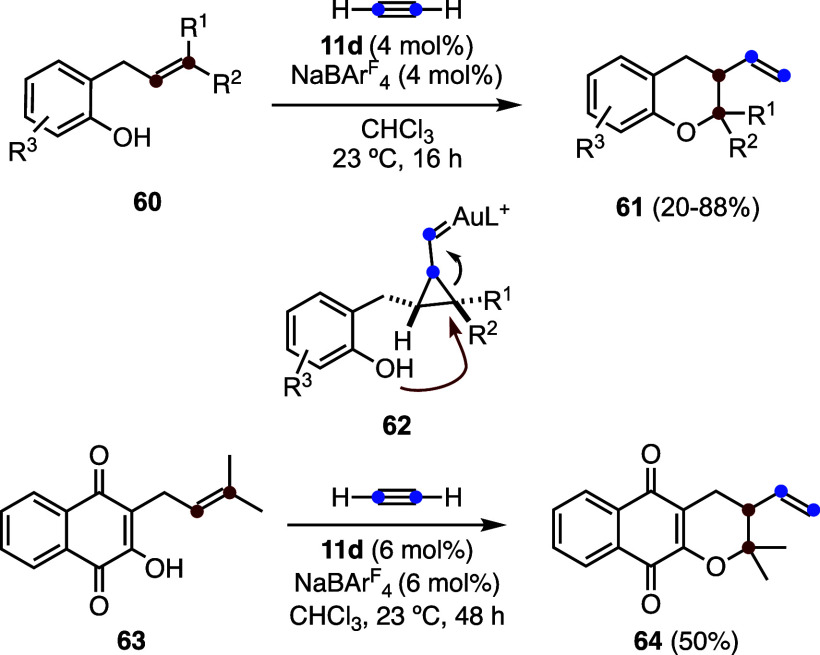
Synthesis of Chromanes by Gold­(I)-Catalyzed Reaction of Acetylene with *o*-Allylphenols

The first fully intermolecular three-component version of the gold­(I)-catalyzed reaction of 1,6-enynes with nucleophiles (**1** to **3** in Scheme 1) was developed with acetylene, electron-rich alkenes **65**, and alcohols to afford β-vinyl hemiaminal scaffolds **66**, by regioselective nucleophilic opening of the cyclopropyl gold­(I) carbene **67** (Scheme 18).^55^ Similarly, biscyclopropane derivative **69** and pyrrolidines or piperidines **71** were obtained by the reaction of acetylene with **68** and **70**, respectively.

**Scheme 18:**
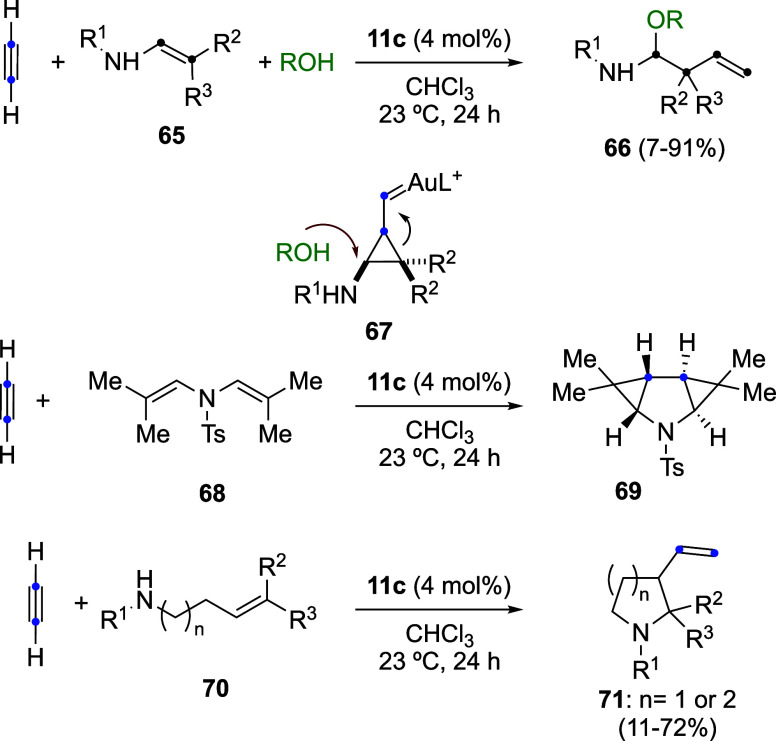
Three-Component Intermolecular Reaction of Acetylene, Alkenes, and Alcohols and Related Transformations

The gold­(I)-catalyzed 1,2-haloalkynylation of alkenes was independently reported by three research groups. Initially, the reaction of chloroalkynes **32a** with alkenes was shown to afford chloroalkynylation products **72** (Scheme 19).^56,57^ Almost simultaneously, bromoalkynylation reactions involving bromophenylacetylene (**73a**) and alkenes were reported, leading to products **74a** and *trans*-homopropargyl bromides **75** and **76** via a 1,2-bromoalkynylation pathway.^58^ In the case of cyclopentene, formation of the corresponding product of [2 + 2] cycloaddition **77** was also observed. In a third study, the scope of the 1,2-haloalkynylation was further expanded using gold­(I) catalysts **78** and **79** (Scheme 19).^59^ In this study, [2 + 2] cycloaddition products analogous to **77** were again detected in a few cases. Notably, chiral catalyst **78** allowed the synthesis of products of type **76** with enantiomeric ratios ranging from 85:15 to 94:6 *er*.^59^

**Scheme 19:**
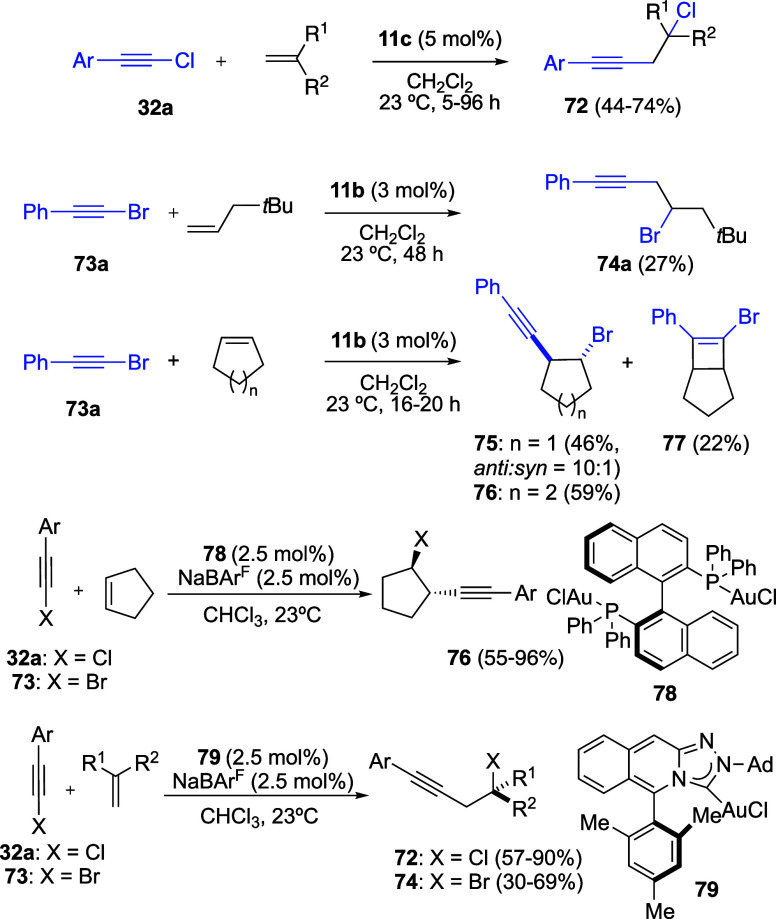
1,2-Haloalkynylation of Alkenes

Reaction between bromoalkynes **73** and allylsilanes **80** exhibits a broad scope, affording a variety of 1,4-enynes **81** with catalyst **11b** (Scheme 20).^58^ Interestingly, in this case, the use of InBr_3_ as a catalyst led to comparable results. Mechanistic insight was gained from isotopic labeling experiments: ^13^C-labeled bromoalkyne **73a-** ^**13**^ **C** reacted with allylsilane **80a** to give **81-** ^**13**^ **C**, demonstrating that the labeled carbon undergoes 1,2-migration from the β- to the α-position relative to the phenyl group. This concealed rearrangement also operates in the 1,2-haloalkynylation process.^58^ Accordingly, the ^13^C-labeled bromoalkyne **73a-** ^**13**^ **C** reacts with cyclohexene to afford **76-** ^**13**^ **C**, further supporting the proposed migratory pathway.

**Scheme 20:**
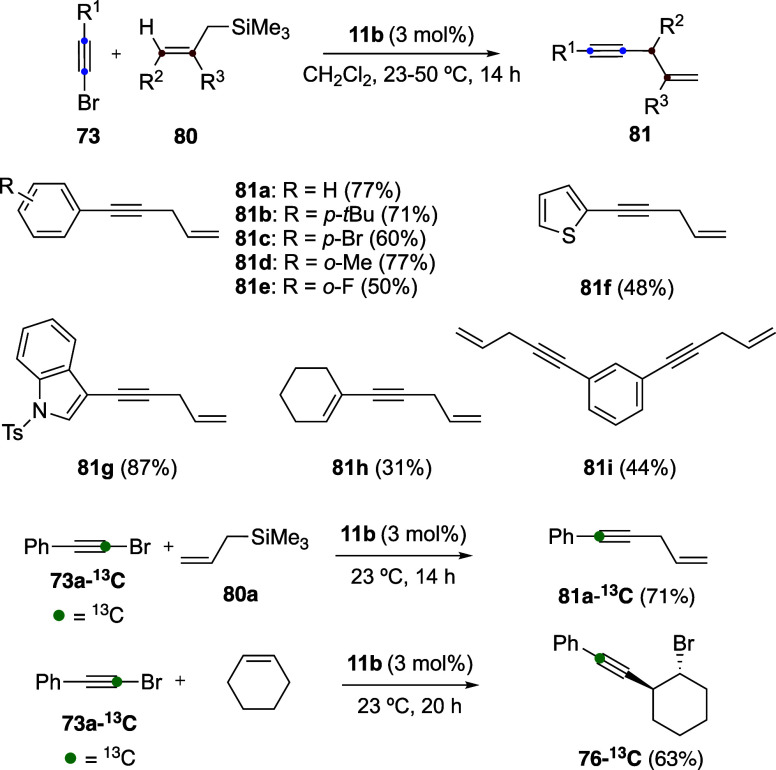
Gold­(I)-Catalyzed Allylation of Alkynes

Accordingly, on the basis of DFT calculations and the ^13^C-labeling experiments, the 1,2-bromoalkynylation and allylation reactions share the same mechanistic pathway (Scheme 21).^58^ In this mechanism, cyclopropyl gold­(I) carbene intermediates **82** undergo an intramolecular attack of Br at the most substituted carbon to generate cyclic bromonium intermediates **83**. Subsequent aryl-assisted opening of **83** leads to the vinylidenephenonium-gold­(I) cation **84**, which ultimately evolves into product **74** or **81a**. This aryl migration closely resembles that observed in the Fritsch–Buttenberg–Wiechell rearrangement.^60^ The alternative opening of **79** to form a gold­(I) vinylidene was also considered as a route for the formation of a 1,2-dihydronaphthalene derivative, obtained from (4-bromobut-3-yn-1-yl)­benzene.^58^ Cyclic bromonium intermediates related to **83** have been also proposed recently in the gold­(I)-catalyzed cyclization of bromo 1,6-enynes.^61^

**Scheme 21:**
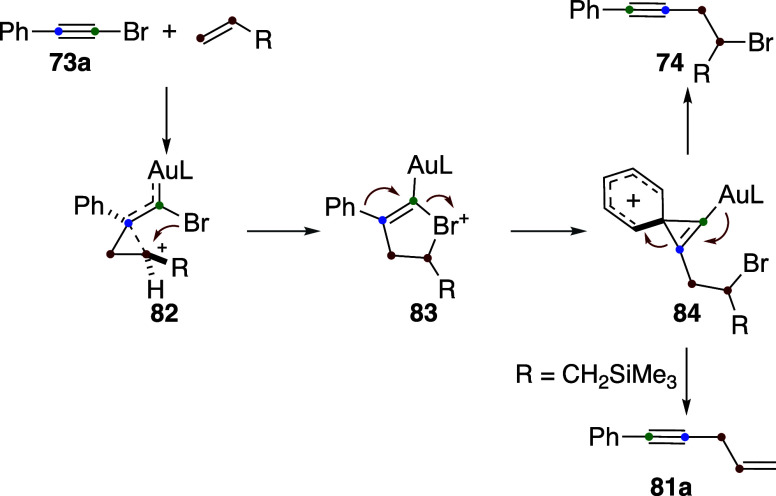
Common Mechanism for the 1,2-Haloalkynylation of Alkenes and the Allylation of Alkynes

Despite the synthetic importance of the phenol synthesis by intramolecular reaction of furans with alkynes **8** and **9**, the development of an intermolecular version was relatively slow. Initially, one example of an intermolecular version was reported between furan **85** and phenylacetylene (**8a**), leading to phenol **86a** in low yield, along with the product of formal hydroarylation **87** under neat conditions in the presence of Schmidbaur–Bayler binuclear gold­(I) complex [(Mes_3_PAu)_2_Cl]­BF_4_ as the catalyst (Scheme 22a).^62,63^ The phenol synthesis follows a rather complex mechanism proceeding via cyclopropyl gold­(I) carbene intermediate **88**, which is followed by opening to form a new gold-carbene **89** (Scheme 22b).^10,11,64^ Cyclization of **89** generates **90** and, subsequently, oxepine **91**, which is in a tautomeric equilibrium with arene oxide **92**. The final substituted phenols **86** are obtained by the opening of the epoxide of **92**. Finally, using NHC-gold­(I) catalysts **52** or **52’**, the intermolecular reaction of furan **85** was carried out with a variety of alkynes **8** to five phenols **86** (Scheme 22c). As an example, a double annulation of **85** with 1,4-diethynylbenzene (**8e**) affords terphenyl derivative **93**.^65^

**Scheme 22:**
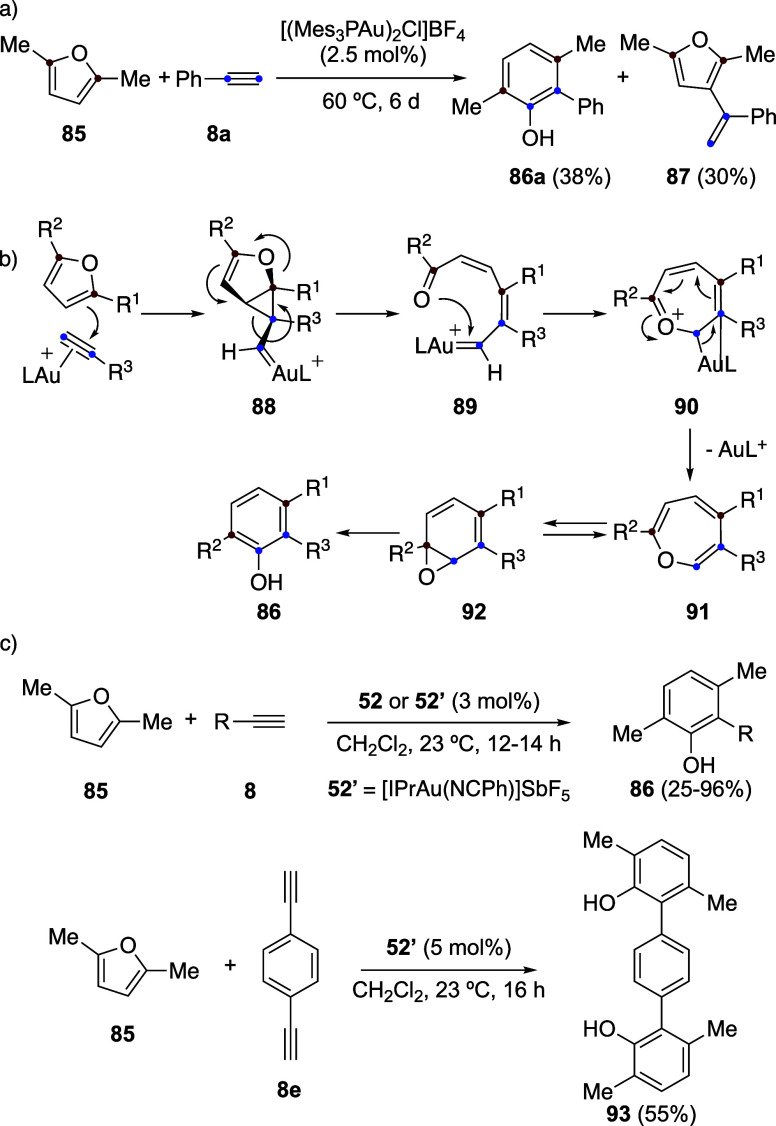
(a) First Example of Intermolecular Gold­(I)-Catalyzed Reaction of Furans with Alkynes. (b) Mechanism of the Intermolecular Reactions of Furans with Alkynes. (c) Intermolecular Reaction of Furans with Alkynes Catalyzed by IPrAu­(I) Complexes

Ynamides **94** are activated upon coordination with gold­(I) to form electrophilic species **95**, which react at C1 with a variety of electrophiles (Scheme 23a). Accordingly, ynamides **94a** react with alkenes to furnish products **96** as a result of a formal (4 + 2) cycloaddition that proceeds via intermediate **97**.^66^ Reaction with more reactive enol ethers leads to either **98** or naphthalenes **99** as products (Scheme 23b).^66^ In the former case, a formal [2 + 2 + 2] cycloaddition takes place, presumably by reaction of a second molecule of enol ether with cyclopropyl gold­(I) carbene **100** to form **101**, which undergoes cyclization to generate six-membered ring **102** and, finally, affording **98**. The formation of naphthalenes can be explained by the elimination of ROH from adducts of type **96**.

**Scheme 23:**
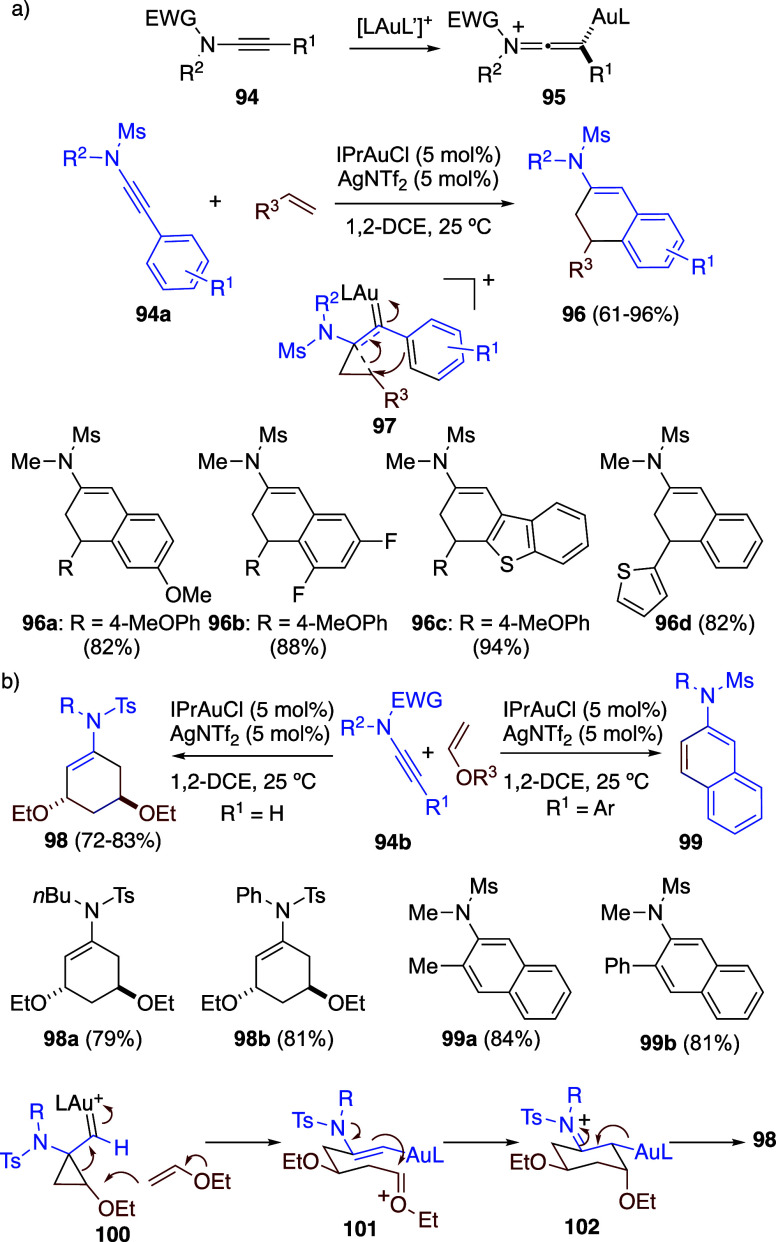
(a) Formal Gold-Catalyzed Intermolecular (4 + 2) Cycloaddition of Ynamides with Alkenes. (b) Gold-Catalyzed Intermolecular Reactions of Ynamides with Enol Ethers

## Reaction of Alkynes with Arenes and Heteroarenes

The gold-catalyzed intermolecular reaction of electron-rich arenes and heteroarenes, commonly referred to as hydroarylation of alkynes or alkenylation of arenes, leading to 1,1-disubstituted alkenes, represents one of the earliest classes of gold­(I)-catalyzed transformations.^14,67–72^ Here, only selected recent developments will be discussed. Intermolecular reactions of furans with alkynes, which proceed through a cyclopropyl gold­(I) carbene intermediate and are therefore closely related to alkyne–alkene reactions, have been covered in the previous section (Scheme 22).

The gold­(I)-catalyzed reaction of 2-alkynyl indoles **103** with alkynes **8** leads to carbazoles **104**, presumably through the initial formation of hydroarylation intermediate **105** (Scheme 24). A hydroarylation is also involved in the synthesis of indolizidines **107** from pyrroles **106** and alkynes **8** (Scheme 25).^73^

**Scheme 24:**
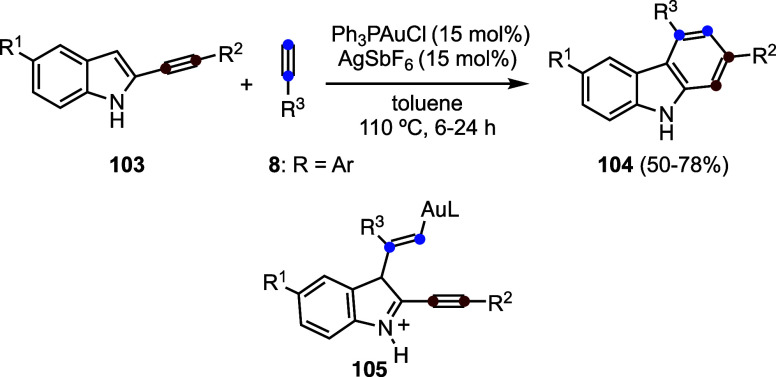
Gold­(I)-Catalyzed Synthesis of Carbazoles

**Scheme 25:**
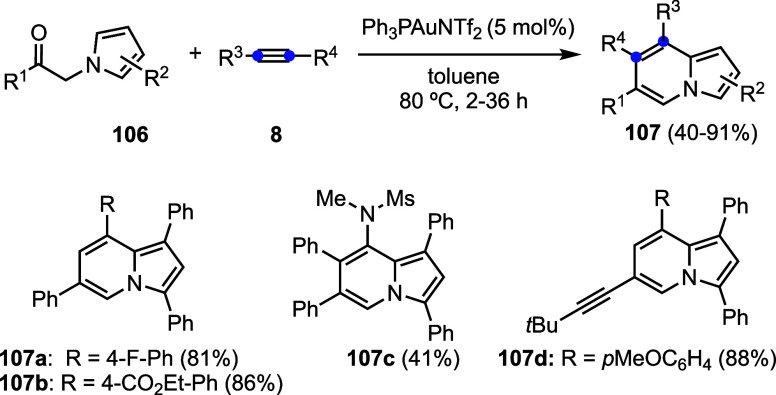
Gold­(I)-Catalyzed Synthesis of Indolizines

The gold­(I)-catalyzed intermolecular addition of electron-rich aromatic compounds to chloroalkynes leads to products **108**, by the *anti*-addition of the arene to the p-alkyne gold­(I) complex (Scheme 26).^74^ The same outcome was observed in the addition of phenols to chloroalkynes to form **109** using gold­(I) catalyst **11d**.^75^ Following previous results in the hydroarylation of indoles,^76,77^ reaction of these highly electron-rich heterocyclic compounds with chloro- and bromoalkynes affords products **110** using gold­(I) catalyst **111** with an electron-donating NHC ligand.^78^ In the case of 3-subsituted indoles, products of 2-alkenylation were obtained in good yields.

**Scheme 26:**
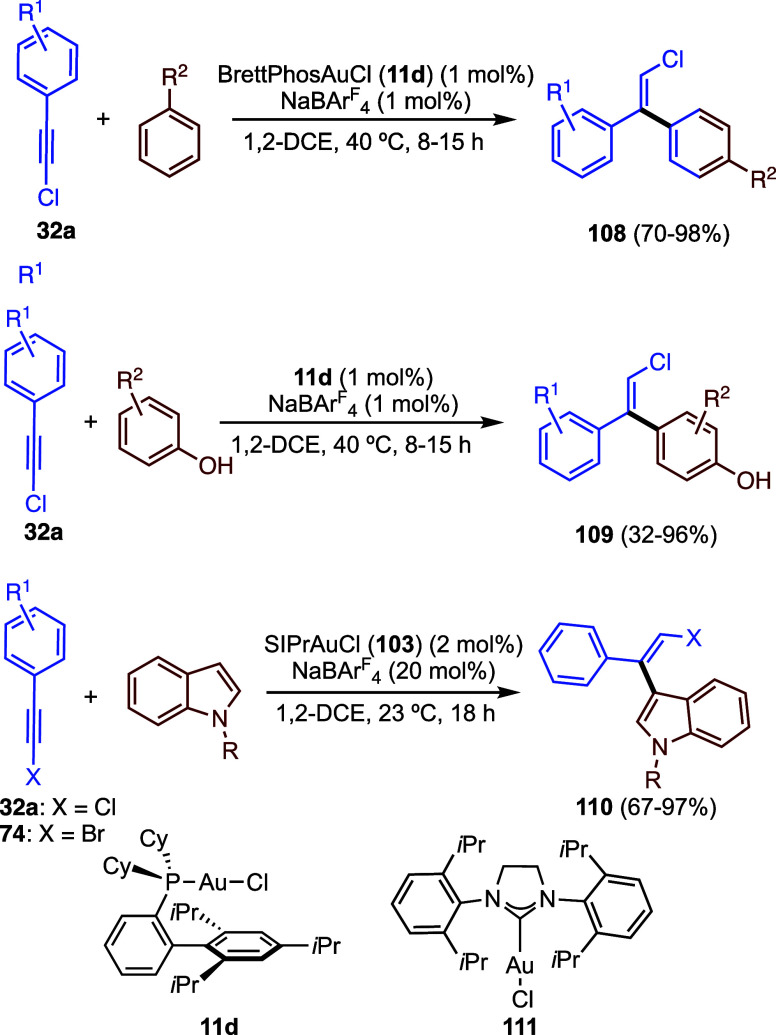
Gold­(I)-Catalyzed *trans*-Addition of Electron-Rich Arenes or Heteroarenes to Halo Alkynes

## Reaction between Two Alkynes

Intermolecular reaction of haloalkynes **32a** and **73a** with aryl alkynes **8** in the presence of catalyst **11c** gives rise to *cis*-haloalkynylation products **112a**–**b** in a general manner (Scheme 27).^79^ Two parallel mechanistic pathways were identified on the basis of DFT calculations and ^13^C-labeling, both initiated by nucleophilic attack of **8** at either C2 or C1 of the chloroalkyne-gold­(I) complex, leading to **112a** and **112a’**, respectively. In the former pathway, intermediate **113a** evolves into five-membered chloronium species **114**, which undergoes a 1,2-aryl shift to afford **112a**. In the alternative pathway, rotation of **113b** enables a 1,3-Cl shift via intermediate **115**, ultimately yielding **112a’**.

**Scheme 27:**
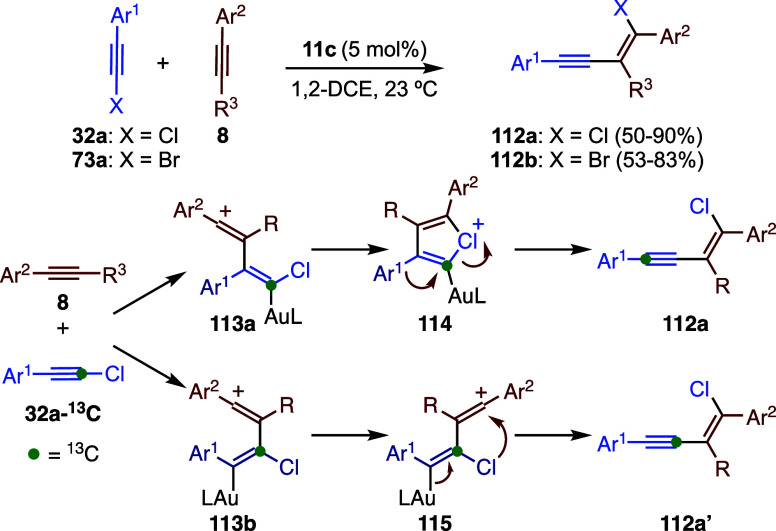
Gold­(I)-Catalyzed Haloalkynylation of Alkynes

Formation of chloronium intermediate **114** followed by 1,2-aryl migration constitutes the predominant pathway (**112a**/**112a’** = 87:13) and closely resembles the mechanism proposed for the 1,2-haloalkynylation of alkenes and the allylation of alkynes, both of which proceed through bromonium intermediates **83** (see Scheme 21).^58^ A similar mechanism to that involved in the formation of products **112a’** via **114** is involved in the head-to-head dimerization of chloro- and bromoalkynes to give rise to 1,2-dihalo-but-1-en-3-ynes.^80^

The gold­(I)-catalyzed haloalkynes with terminal alkynes proceed through divergent pathways depending on the nature of the counteranion of the gold­(I) catalyst (Scheme 28).^81^ Reaction of haloalkynes **32a** and **73a** with alkynes **8** leads to *cis*-bromoalkynylation products **108** using catalytic system **111**/NaBArF_4_, following the pathway outlined before for the formation of products **116** (Scheme 27).^79^ On the other hand, when weakly basic AgOTf was used as an activator of gold­(I) catalyst **111**, *trans*-hydroarylation products **117** were obtained. The formation of this new class of products was rationalized by an alkynylation of the haloalkyne that is concerted with the deprotonation by the weakly basic triflate counteranion in transition state **118**, leading to intermediate **119**, which then undergoes protodeauration to afford **117**. Consistent with a transition state of type **118** for the turnover determining state in the catalytic cycle, a primary kinetic isotope effect of *k*_H_/*k*_D_ = 2.4 was determined in the hydroalkynylation reaction with **111**/AgOTf. Triflate acts as a proton shuttle, assisting the deprotonation that is concerted with C–C bond formation and allowing a favorable exchange equilibrium with the activated haloalkyne. In addition to bromoalkynes **73a**, the reaction was also performed with chloro- and iodophenylacetylene.

**Scheme 28:**
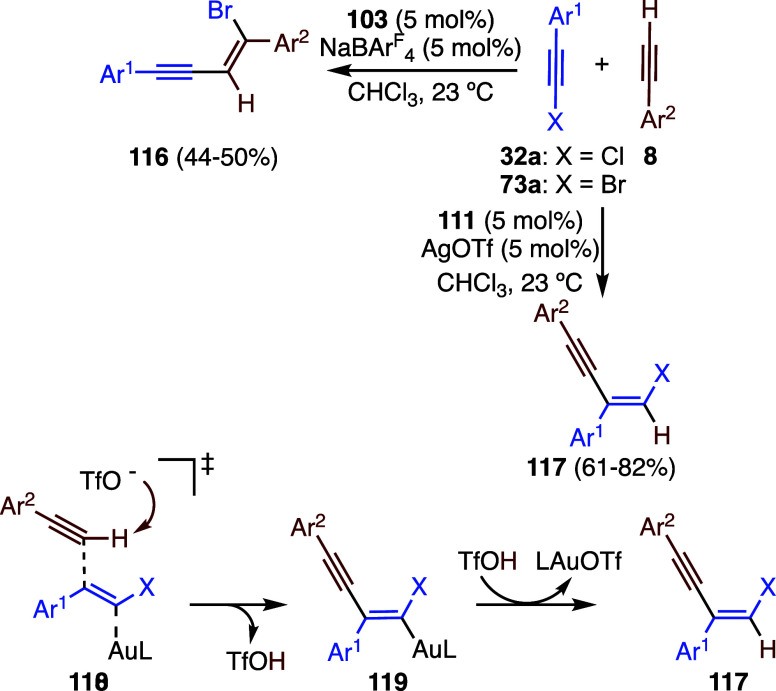
Gold­(I)-Catalyzed Haloalkynylation of Alkynes

Oxazolidinone ynamides **120** undergo hydroalkynylation with terminal alkynes **8** to form *trans*-configured 1,3-enynes **121** (Scheme 29).^82^ A mechanistic pathway distinct from that computed for the gold­(I)-catalyzed haloalkynylation of alkynes (Scheme 26)^79^ was proposed here. Accordingly, a dual gold­(I)-catalyzed process is achieved by nucleophilic attack of σ-alkyne-gold­(I) complex **122** to π-alkynylgold­(I) **123** to form intermediate **124**, which generates **121** by protodeauration. Dual gold­(I)-catalysis has also been proposed in the dimerization of haloalkynes that leads to *gem*-dihalogenated 1,3-enynes by formal head-to-tail coupling.^83^

**Scheme 29:**
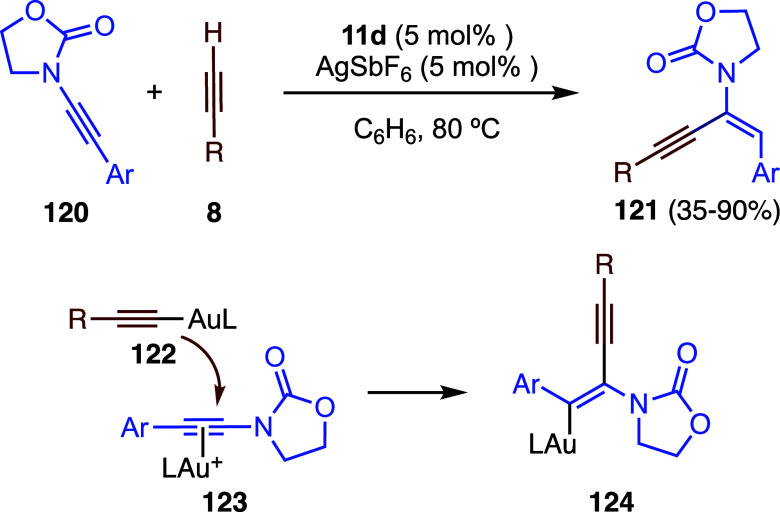
Gold-Catalyzed Intermolecular Reaction of Ynamides with Alkynes

Ynamides can act as nucleophiles toward p-ynamide-gold­(I) complexes, leading to dimerization reactions (Scheme 30).^84^ Thus, ynamides **125a** react under gold­(I) catalysis to afford cyclopentadienes **126** via intermediates **127**, which undergo a 1,5-H shift to form **120**, followed by a Nazarov-type cyclization to generate gold­(I) carbene **129**. A subsequent 1,2-H shift then furnishes **126**. Substrates **125b** bearing cycloalkyl substituents also give rise to tricyclic compounds **130**. However, in this case, intermediates of type **131** undergo intramolecular C–H insertion to form a cyclopropane ring, thereby constructing the tricyclic framework of **130**. Oxazolidinone-derived ynamides **120** (see Scheme 29) likewise undergo gold­(I)-catalyzed dimerization. However, this process is largely unselective leading to naphthalene compounds in moderate yields, along with other products.^84^

**Scheme 30:**
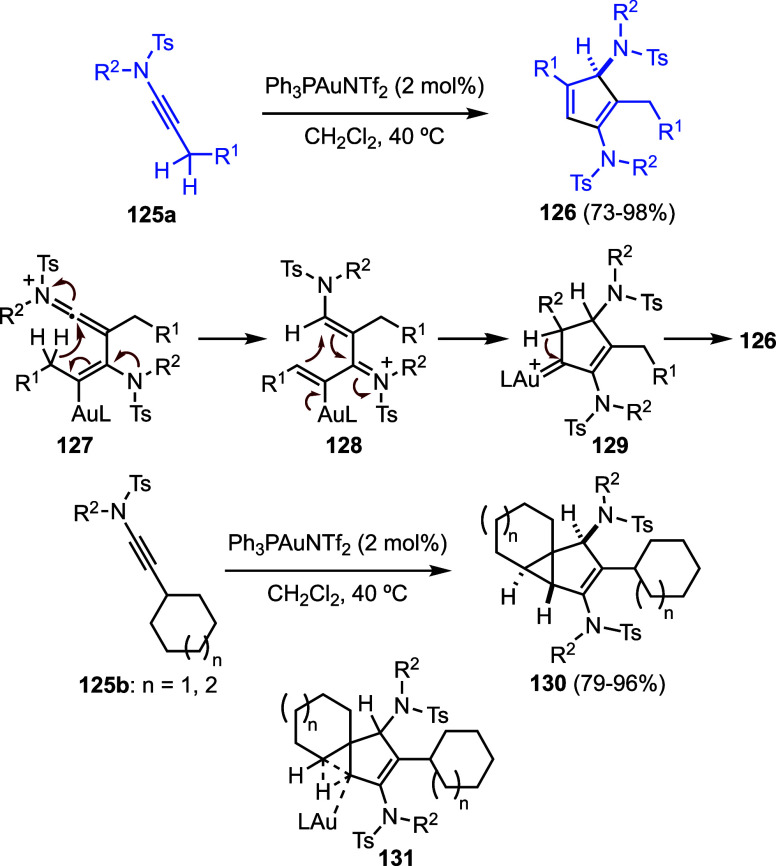
Gold-Catalyzed Dimerization of Ynamides

## Reactions of Other *C*-Nucleophiles with Alkynes

Two research groups independently developed a novel synthesis of furans based on the gold­(I)-catalyzed addition of sulfur ylides to terminal alkynes (Scheme 31). In this process, reaction of ylides **132** with terminal alkyl-substituted alkynes **8** affords 2,4-disubstituted furans **133**.^85^ Transformation of the initial adduct **134** is followed by sulfide elimination to generate gold­(I) carbene **135**, which undergoes cyclization to give **136**. Finally, formal LAu^+^ elimination furnishes **133**. Similarly, aryl-substituted alkynes **8** react with sulfur ylides **137** in the presence of gold­(I) catalysts **11d**–**e** to furnish 2,3,4-trisubstituyted furans **138** by the same mechanism.^86^

**Scheme 31:**
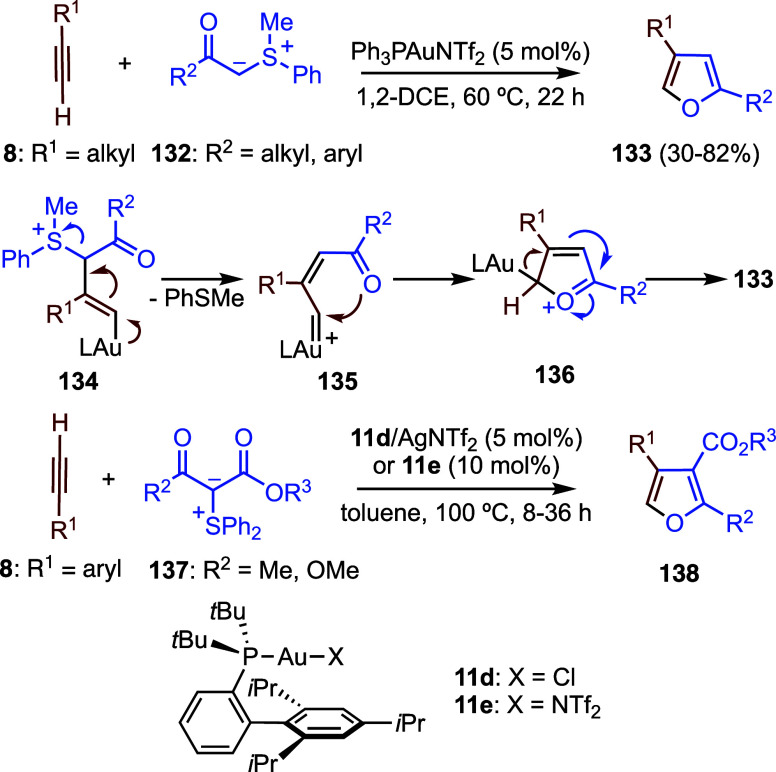
Gold­(I)-Catalyzed Synthesis of Furans from Sulfur Ylides

The addition of 1,3-dicarbonyl compounds to alkynes, originally developed with In­(III) catalysts,^87,88^ can also be carried out by using gold­(I) and Ga­(III) to form 2-alkenylated 1,3-dicarbonyl compounds.^89^ Gold­(I) catalysts generated in situ by reaction of LAu-Pht (Pht = phthalimide) with Bro̷nsted acids can also catalyze this reaction.^90^ An enantioselective intermolecular addition of β-ketoamides **139** to 1-alkynes **8** has been achieved using chiral *N*,*N*′-dioxide-indium­(III) or nickel­(II) Lewis acid and achiral gold­(I) complexes to from products of type **140** (Scheme 32).^91^ In addition to β-ketoamides **139**, β-ketoesters, and 1,3-dicarbonyl compounds are also substrates of this reaction.

**Scheme 32:**
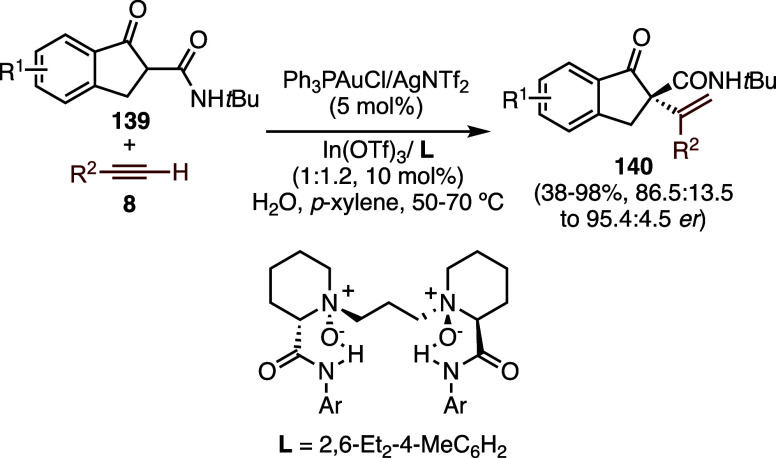
Gold­(I)- and In­(III) Enantioselective Reaction of β-Ketoamides with Alkynes

## Conclusions and Outlook

Most of the pathways outlined for gold­(I)-catalyzed intramolecular reactions of 1,*n*-enynes shown in Scheme 1 have been translated into intermolecular reactions (Scheme 33). Thus, there are several examples of partially intermolecular additions of nucleophiles to alkynes and alkenes to form products of type **I**, in which the nucleophiles are attached to the alkyne (see: Scheme 10,^38,41^ Scheme 11,^42,43^ Scheme 23 ^66^) or the alkene (see Scheme 17).^54^ A fully intermolecular, three-component reaction has also been developed (Scheme 18).^55^ The 1,2-haloalkynylation of alkenes (Scheme 19)^56–59^ and the allylation of alkynes (Scheme 20)^58^ may be considered as variations of reactions leading to products of type **I**. Importantly, in these reactions, five-membered ring chloronium or bromonium intermediates have been detected by isotopic labeling and DFT calculations.

**Scheme 33:**
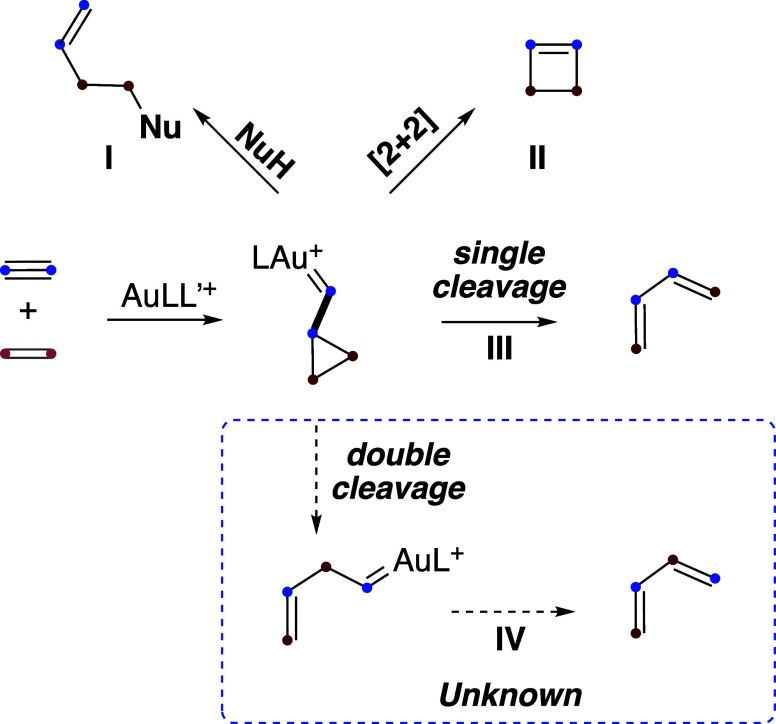
Intermolecular Versions of Cyclizations of 1,*n*-Enynes

Formation of cyclobutenes **II** (Scheme 32) has been found to be the major pathway in reactions of both electron-rich (see Schemes 2, 4, and 5)^29–31^ and electron-deficient alkynes (see Scheme 8,^37^ Scheme 11 ^42,44^ ). Ynol ethers also react with alkenes to give cyclobutenes **II** (see Scheme 9).^38^

Rearrangement via single-cleavage-type reactions leading to dienes **III** has also been reported with both electron-rich (see Scheme 6 ^30^) and electron-deficient alkynes (Scheme 12 ^42,43^ ). Acetylene also reacts to give rise to 1,3-dienes **III** in reactions with a few alkenes (Scheme 14).^49^ In contrast, no examples have been reported to date of a double-cleavage rearrangement leading to products of type **IV** (Scheme 32). Accordingly, it may be worthwhile to explore systems that favor this pathway, as the corresponding 1,3-diene precursors are allyl gold­(I) carbene intermediates that could potentially be intercepted intermolecularly by alkenes or other reaction partners.

Studies on the formation of 1,3-butadienes single-cleavage-type reactions^30,42,43,49^ show that the regioselectivity in the formation of cyclopropyl gold­(I) intermediates depends on the substitution pattern of the alkyne (Scheme 34). Specifically, for terminal alkynes with electron-donating (EDG) substituents, the gold­(I) carbene is preferentially generated at the alkyne carbon located β to electron-donating groups, whereas formation at the α position relative to electron-withdrawing (EWG) groups is favored, leading to intermediates **10** and **43**, respectively.

**Scheme 34:**
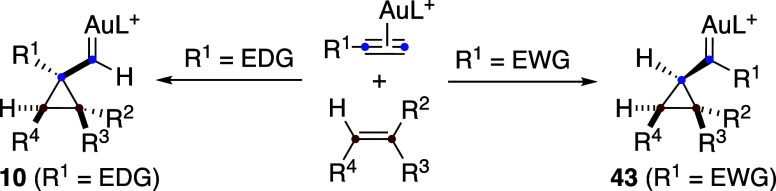
Dependence of the Regioselectivity on the Nature of the Alkyne in the Formation of Cyclopropyl Gold­(I) Carbenes

Although a wide variety of alkenes have been studied as partners in these reactions, notably absent are allenes, with a notable exception (product **40e** in Scheme 11 ^42^). Considering that cyclizations of allenynes can either proceed with the allene reacting with a gold­(I)-π-alkyne or with the alkyne attacking the gold­(I)-π-allene,^92–97^ exploration of intermolecular alkyne–allene reactions may uncover interesting novel transformations of great synthetic value.

Transformations in which the alkyne is oxidized through the addition of a pyridine *N*-oxide, sulfoxide, or related reagent to generate an α-oxo gold­(I) carbene intermediate,^98–100^ fall outside the scope of this Perspective. However, it is also worth noting that intermolecular transformations of α-oxo gold­(I) carbenes involving *C*-nucleophiles remain relatively rare.

Despite the challenges imposed by the linear coordination of gold­(I) complexes and the very small size of alkynes,^101,102^ enantioselective transformations have been developed in the [2 + 2] cycloaddition to form cyclobutenes (see Schemes 4 and 5 ^34^), the formal (4 + 2) annulation of propiolates and alkenes (Scheme 11 ^44^), and a limited of other cases (Scheme 17 ^54^ and Scheme 19 ^59^). However, enantioselectivities in the range of 93:7 to 94:6 *er* have been achieved only in a few cases. Consequently, there remains substantial room for improvement to attain competitive levels of enantioselectivities in intermolecular processes involving inexpensive, commercially available, or readily accessible alkynes and alkenes, thereby enabling these transformations to become commonplace in the repertoire of organic synthesis.
